# Fibroblasts derived from human embryonic stem cells direct development and repair of 3D human skin equivalents

**DOI:** 10.1186/scrt51

**Published:** 2011-02-21

**Authors:** Yulia Shamis, Kyle J Hewitt, Mark W Carlson, Mariam Margvelashvilli, Shumin Dong, Catherine K Kuo, Laurence Daheron, Christophe Egles, Jonathan A Garlick

**Affiliations:** 1 Program in Cell, Molecular and Developmental Biology, Sackler School of Graduate Biomedical Sciences, Tufts University School of Medicine, 136 Harrison Avenue, Boston, MA, 02111, USA; 2 Department of Oral and Maxillofacial Pathology, Tufts University School of Dental Medicine, 1 Kneeland street, Boston, MA, 02111, USA; 3 Department of Biomedical Engineering, Tufts University, 4 Colby street, Medford, MA, 02155, USA; 4 Massachusetts General Hospital, Center for Regenerative Medicine and Technology, 185 Cambridge Street, Boston, MA, 02114, USA

## Abstract

**Introduction:**

Pluripotent, human stem cells hold tremendous promise as a source of progenitor and terminally differentiated cells for application in future regenerative therapies. However, such therapies will be dependent upon the development of novel approaches that can best assess tissue outcomes of pluripotent stem cell-derived cells and will be essential to better predict their safety and stability following *in vivo *transplantation.

**Methods:**

In this study we used engineered, human skin equivalents (HSEs) as a platform to characterize fibroblasts that have been derived from human embryonic stem (hES) cell. We characterized the phenotype and the secretion profile of two distinct hES-derived cell lines with properties of mesenchymal cells (EDK and H9-MSC) and compared their biological potential upon induction of differentiation to bone and fat and following their incorporation into the stromal compartment of engineered, HSEs.

**Results:**

While both EDK and H9-MSC cell lines exhibited similar morphology and mesenchymal cell marker expression, they demonstrated distinct functional properties when incorporated into the stromal compartment of HSEs. EDK cells displayed characteristics of dermal fibroblasts that could support epithelial tissue development and enable re-epithelialization of wounds generated using a 3D tissue model of cutaneous wound healing, which was linked to elevated production of hepatocyte growth factor (HGF). Lentiviral shRNA-mediated knockdown of HGF resulted in a dramatic decrease of HGF secretion from EDK cells that led to a marked reduction in their ability to promote keratinocyte proliferation and re-epithelialization of cutaneous wounds. In contrast, H9-MSCs demonstrated features of mesenchymal stem cells (MSC) but not those of dermal fibroblasts, as they underwent multilineage differentiation in monolayer culture, but were unable to support epithelial tissue development and repair and produced significantly lower levels of HGF.

**Conclusions:**

Our findings demonstrate that hES-derived cells could be directed to specified and alternative mesenchymal cell fates whose function could be distinguished in engineered HSEs. Characterization of hES-derived mesenchymal cells in 3D, engineered HSEs demonstrates the utility of this tissue platform to predict the functional properties of hES-derived fibroblasts before their therapeutic transplantation.

## Introduction

The use of pluripotent, human stem cells, including human embryonic stem (hES) cells and human induced pluripotent stem (hiPS) cells, for future therapies provides advantages over more traditional sources of progenitor cells, such as adult stem cells, due to their ability to give rise to a variety of differentiated cell types and to their unlimited expansion potential [1,2]. However, such therapies will be dependent upon the development of novel approaches that can best assess tissue outcomes of hES- and hiPS-derived cells and will be essential to better predict their safety and stability following *in vivo *transplantation. One possible approach would be to use three dimensional (3D), engineered tissues to monitor the functional outcomes of hES- and hiPS-derived cells. By providing an *in vivo*-like microenvironment that enables progenitor cells to manifest their *in vivo *characteristics in 3D tissue context, tissue engineering can play an important role in determining the function, stability, and safety of hES- and hiPS-derived cells before their future application.

Stromal fibroblasts play a critical role in regulating tissue homeostasis and wound repair through the synthesis of extracellular matrix proteins and by secreting paracrine-acting growth factors and cytokines that have a direct effect on the proliferation and differentiation of adjacent epithelial tissues [3-6]. Despite the critical impact of this reciprocal cross-talk between stromal fibroblasts and epithelial cells on tissue homeostasis, little is known about the identity and maturational development of the precursor cells that give rise to these fibroblasts. This incomplete understanding of fibroblast lineage development is in large part due to the lack of definitive markers and to their cellular heterogeneity *in vivo *that has complicated their isolation, characterization, and potential therapeutic applications [7-9].

In light of this, human pluripotent stem cells may serve as an alternative to adult tissues of more uniform fibroblasts that may provide more predictable tissue outcomes upon their therapeutic use. Several previous studies have demonstrated the derivation of mesenchymal stem cell (MSC)-like cells from hES cells that can differentiate to bone, fat, and cartilage [10-13], and fibroblast-like cells that have been used as autogenic feeders to support the culture of undifferentiated hES cells [14-17]. In our previous work, we have demonstrated that hES cells give rise to fibroblast-like cells [18]; however, we have not determined if hES-derived cells can manifest the functional properties of dermal fibroblasts that can support the organization and development of 3D skin-like tissues also known as human skin equivalents (HSEs) through epithelial-mesenchymal cross-talk. As the morphogenesis, homoeostasis, and repair of many tissues depends on interactions between epithelial cells and their adjacent stromal fibroblasts [3-6], the functional analysis of hES-derived fibroblasts could best be accomplished in such engineered HSEs that demonstrate many features of their *in vivo *counterparts.

In this study, we have characterized two cell lines with features of MSC lineages (EDK and H9-MSC) that differ from each other in their production of hepatocyte growth factor (HGF), a growth factor known to be secreted by dermal fibroblasts that supports epithelial development and repair. In monolayer cultures, we found that EDK and H9-MSCs exhibited considerable overlap as seen by their mesenchymal morphology and expression of surface markers characteristic of both MSCs and dermal fibroblasts. However, EDK cells could not undergo differentiation to bone and fat and demonstrated properties similar to stromal fibroblasts that could support epithelial tissue development and enable re-epithelialization of HSEs that was linked to the elevated expression and secretion of HGF. In contrast, H9-MSCs displayed multipotent differentiation capacity typical of an MSC phenotype [19], but did not support epithelial tissue development or repair, possible due to a low level of HGF production. When HGF secretion from EDK cells was suppressed by shRNA, epithelial repair was significantly decreased, suggesting that the regenerative phenotype of EDK cells is mediated, at least in part, by HGF secretion. HSEs used in our studies provided a complex tissue microenvironment that enabled characterization of the functional properties of hES-derived fibroblasts and provide an important platform to further establish their stability, safety, and efficacy for future therapeutic transplantation.

## Materials and methods

### Monolayer cell culture of hES cells and directed differentiation to mesenchymal and fibroblast fates

**Figure 1:**
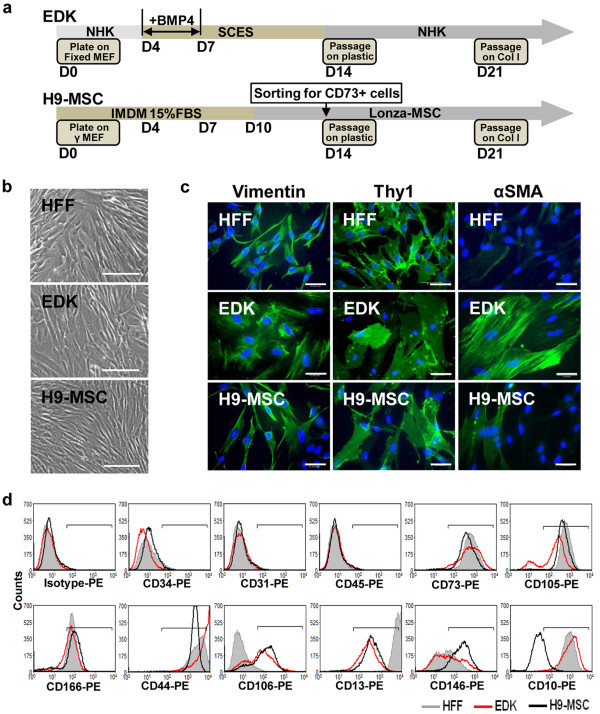
**Characterization of hES-derived mesenchymal cell lines**. **(a) **Schematic summarizing the two protocols used for generating EDK and H9-MSC from H9 human embryonic stem (hES) cells. **(b) **Cellular morphology of human foreskin fibroblasts (HFF), EDK, and H9-MSC using phase contrast. Bars, 50 μm. **(c) **Expression of mesenchymal markers vimentin, Thy1, and α-SMA following immunofluorescence staining. Bars, 50 μm. **(d) **Comparison of surface antigen expression on HFF, EDK, and H9-MSC by flow cytometric analysis. EDK and H9-MSCs express markers associated with mesenchymal phenotype (positive for expression CD73, CD105, CD44, CD13, CD106, and CD166; negative for expression of CD45, CD34, and CD31). EDK and H9-MSCs show significant differences in expression of CD146 and CD10 antigens. (HFF, filled grey profile; EDK, red profile; H9-MSC, black profile).

A H9 hES cell line was maintained in culture as described [20], on irradiated feeder layers of mouse embryonic fibroblasts (MEF). EDK cells were prepared using our previously described protocol [18]. Briefly, we derived multiple, independent EDK cell lines by first growing H9 hES cells on MEFs fixed in 4% formaldehyde in differentiation medium supplemented with 0.5 nM human BMP-4 (R&D Systems, Minneapolis, MN, USA) from day four to seven of differentiation. These EDK cell lines were then propagated first on tissue culture plastic and then expanded on type I collagen-coated plates (BD Biosciences, San Jose, CA, USA). EDK cell lines were then screened by ELISA to determine their secretion of HGF and KGF and the lines with highest production of these growth factors were chosen for further study. H9-MSCs were prepared as previously described [21]. Briefly, H9 hES cells were grown on irradiated feeder layers of MEFs in differentiation medium for 10 days and then switched to MSC medium (Lonza, Basel, Switzerland) for four extra days to enrich for MSC-like cells. Differentiating cells were then fluorescence-activated cell sorting (FACS)-sorted for CD73-positive cells expanded on MSC medium first on tissue culture plastic and then passaged on type I collagen-coated plates. Both protocols for cell derivation from H9 hES cells are summarized in (Figure 1a). Control human dermal fibroblasts (HFF) were derived from newborn foreskin and grown in DMEM (Invitrogen, Carlsbad, CA, USA) supplemented with 10% FBS (Hyclone, Logan UT, USA).

### Construction of human skin equivalents and 3D tissue model of cutaneous wound healing

Engineered 3D HSEs were constructed as previously described [22] by adding HFF, EDK, or H9-MSCs or no cells into type I collagen gels (Organogenesis, Canton, MA, USA) to a final concentration of 2.5 × 10^4 ^cells/ml. Human keratinocytes (NHK) at a concentration of 5 × 10^5 ^were seeded onto the collagen matrix and tissues were then maintained submerged in low calcium epidermal growth media for two days, for an additional two days in normal calcium media, and raised to the air-liquid interface (Organogenesis, Canton, MA, USA). For the preparation of 3D tissue models of cutaneous wound healing, HSEs constructed using HFF were wounded with a 4 mm punch and placed on the surface of a contracted collagen gel populated with either HFF, EDK, H9-MSCs, or without cells as previously described [22,23]. Wounded cultures were maintained at the air-liquid interface for 72 or 96 hours at 37°C in 7.5% CO_2 _to monitor re-epithelialization. For quantitative analysis of wound re-epithelialization, tissue samples were fixed in 4% neutral-buffered formalin, embedded in paraffin, and serially sectioned at 8 μm. Histological sections were stained with H&E, images were captured using a Nikon Eclipse 80i microscope (Nikon Instruments Inc., Melville, NY, USA) equipped with a SPOT RT camera (Diagnostic Instruments, Sterling Heights, MI, USA) and analysed using Spot Advanced software (Diagnostic Instruments, Sterling Heights, MI, USA).

### Real-time RT-PCR

Total RNA was extracted from confluent cultures of EDK, H9-MSC, or HFF cells using RNeasy kit (Qiagen, Valencia, CA, USA) and cDNA was transcribed with 1 μg RNA using the Quantiscript Reverse Transcriptase (Qiagen, Valencia, CA, USA). For each real-time RT-PCR reaction, we used 20 ng of cDNA, 200 nM of each primer, and 2X SYBRgreen (Applied Biosystems, Foster City, CA, USA) at a total sample volume of 12.5 μL and samples were run in triplicates on the Bio-Rad CFX96 Real-Time PCR Detection System according to manufacturers' instructions. The relative level of gene expression was assessed using the 2 ^-ΔΔC^t method and results are presented as an average of two experiments and three technical replicates. The following oligonucleotide primer sequences were used: GAPDH-F1: 5'-tcgacagtcagccgcatcttcttt-3', GAPDH-R1: 5'-accaaatccgttgacctt-3', HGF-F1: 5'-aggggcactgtcaataccatt-3', and HGF-R1: 5'-cgtgaggatactgagaatcccag-3'.

### RNAi

The pLKO.1-puro non-target shRNA control vector and the pLKO.1-puro vector containing shRNA against HGF (TRCN clone 3310) were purchased from Sigma (Sigma, MISSION shRNA, St. Louis, MO, USA). Lentiviral particles were generated in 293FT cells using ViraPower™ Lentiviral Expression System (Invitrogen, Carlsbad, CA, USA) according to manufacturer protocol. Knockdown viruses were titered using an end-point dilution assay and cell lines were infected with lentiviruses at a multiplicity of infection of one. Stable cell lines were selected with puromycin (2 μg/ml).

### ELISA

Tissue culture supernatants were harvested and processed using commercial DuoSet HGF ELISA kit (R&D Systems, Minneapolis, MN, USA). Media was assayed in triplicates from at least three independent samples. For monolayer cultures, the values were normalized according to cell numbers counted in the respective cultures at the time of supernatant harvesting and expressed in pg/ml per 10^4 ^cells.

### Antibody-based cytokine array

HFF, EDK, or H9-MSCs 10^6 ^cells were seeded onto 100 mm tissue culture plates and grown in 10 ml of tissue culture medium. Forty-eight hours before the analysis, cultures were maintained in 5 ml of tissue culture medium. Tissue culture supernatants were harvested and the supernatants from the plates containing equal cell numbers were processed using commercial Proteome Profiler Human Angiogenesis Antibody Array (R&D Systems, Minneapolis, MN, USA) according to manufacturer protocol. Histogram profiles for select analytes were generated by quantifying the mean spot pixel densities from the array membrane using ImageJ software (U. S. National Institutes of Health, Bethesda, Maryland, USA).

### Morphologic, immunofluorescent, and immunohistochemical analysis of human skin equivalents

HFF, EDK, or H9-MSCs were grown to confluence on glass or type I collagen-coated coverslips, fixed in 4% paraformaldehyde, and permeabilized using 0.1% triton X-100. Cells were stained using primary monoclonal antibodies directed against vimentin (Abcam, Cambridge, MA, USA), α-SMA (Chemicon, Temecula, CA, USA), Thy-1 (Calbiochem, San Diego, CA, USA) followed by Alexa 488-conjugated goat anti-mouse secondary antibodies (Invitrogen, Carlsbad, CA, USA) for one hour. For frozen section analysis, tissues were frozen in O.C.T. compound (Sakura Finetek USA, Torrance, CA, USA) and 8 μm sections were fixed in 4% paraformaldehyde and immunostained using primary antibodies directed against K1/10 (Abcam, Cambridge, MA, USA) followed by Alexa 594-conjugated goat anti-mouse secondary antibodies (Invitrogen, Carlsbad, CA, USA). For morphologic analysis, H&E staining was performed on paraffin-embedded tissues sectioned at 8 μm thickness. For proliferation analysis, HSEs were labeled with a six-hour pulse of BrdU (Sigma, St. Louis, MO, USA) at a final concentration of 10 μM. Immunohistochemical staining was performed on paraffin-embedded tissues sectioned at 8 μm thickness using monoclonal antibodies against BrdU (Roche, Indianapolis, IN, USA) and Vectastain ABC (peroxidase) kit (Vector Labs, Burlingame, CA, USA). The number of BrdU-positive cells was determined and expressed as a percentage of total cells in the basal layer. Two independent experiments with a minimum of three observations for each condition were analyzed.

### Flow cytometric analysis of hES-derived cell lines

HFF, EDK, or H9-MSCs were grown to confluence, trypsinized, pelleted, and resuspended in 2% FBS in PBS solution. Cell suspensions were mixed with 15 μl of PE-conjugated anti -CD73, -CD10, -CD13, -CD105, -CD106, -CD166, -CD44, -CD34, -CD45, -CD31 or isotype control-IgG1k (BD Pharmingen, San Jose, CA, USA), and flow cytometric analysis was performed using a FACSCalibur (BD Biosciences, San Jose, CA, USA) and analyzed using CellQuest (BD Biosciences, San Jose, CA, USA) and Summit V4.3 software (Dako, Carpenteria, CA, USA).

### Osteogenic and adipogenic differentiation assays

To induce osteogenesis HFF, EDK, or H9-MSCs were grown to confluence in the presence of 100 nM dexamethasone, 0.05 mM ascorbic acid, and 10 mM-glycerophosphate (all from Sigma, St. Louis, MO, USA) in DMEM supplemented with 1% of non-essential amino-acids (Invitrogen, Carlsbad, CA, USA), and 10% FBS. After 28 days, cultures were fixed in 1% formaldehyde, and stained for 10 minutes with alizarin red solution (Sigma, St. Louis, MO, USA) to determine calcium deposition, and staining was quantified spectrophotometrically (A_550_) after solubilization with 10% CPC (Sigma, St. Louis, MO, USA). To induce adipogenesis HFF, EDK, or H9-MSCs were grown to confluence in 24-well plates in the presence of 1 μM dexamethasone, 50 μM indomethacin, 5 μg/ml insulin, and 0.5 mM 3-isobutyl-1-methyl-xanthine (all from Sigma, St. Louis, MO, USA) in DMEM supplemented with 1% of non-essential amino-acids, and 10% FBS. After 28 days, cultures were fixed in 4% formalin and stained with oil red O solution (Sigma, St. Louis, MO, USA) for 30 minutes to visualize oil droplets, and staining was quantified spectrophotometrically (A_500_) after solubilisation with isopropanol. All results are presented as mean +/- standard deviation (SD) of three experiments and three technical replicates.

### Statistical analysis

Data are expressed as mean ± SD of at least three independent samples. Statistical comparisons between groups were performed with a two-tailed Student's t-test, *P ≤ 0.05 and **P ≤ 0.01 was considered significant.

## Results

### EDK and H9-MSC generated from hES cells demonstrate morphologic and phenotypic characteristics of mesenchymal cells

We generated two populations of mesenchymal cells (EDK and H9-MSC) from hES cells using previously established protocols as shown schematically (Figure 1a). EDK cells were derived using a differentiation protocol that we had previously established to generate fibroblast-like cells from H9 hES cells [18]. To ensure the fibroblast-like phenotype of EDK cells, several cell lines were derived from hES-cells using the same differentiation conditions and were screened for the secretion of paracrine factors known to be secreted at high levels in dermal fibroblasts keratinocyte growth factor (KGF) and hepatocyte growth factor (HGF); data not shown) [5,6]. Only EDK cell lines secreting elevated levels of KGF and HGF were selected for further study. H9-MSCs were derived using an alternative differentiation protocol that has previously been shown to generate MSC-like cells from H9 hES cells based on FACS-isolation of CD73-positive cell subpopulations [21]. Both EDK and H9-MSC populations showed similar fibroblast morphology and expression of Thy-1 and vimentin by immunohistochemical staining that were similar to that seen in dermal HFF, while α-SMA-positive cells were observed more frequently in EDK cultures than in either H9-MSC or HFF (Figures 1b and 1c). Surface marker profiling of EDK and H9-MSCs was performed by flow cytometric analysis and compared with the profile generated for HFFs. EDK and H9-MSC lines presented a similar profile of surface antigen expression characteristic of mesenchymal cells. Both hES-derived cell lines expressed CD73, CD105, CD106, CD44, CD166, CD13, and CD146 and lacked expression of hematopoietic and endothelial lineage markers CD45, CD34, and CD31 (Figure 1d). The intensity of fluorescent labelling and the distribution of labelled cells were similar for CD73, CD105, CD106, CD44, CD166, and CD13 when EDK and H9-MSC were compared. However, expression of CD10 was significantly higher, while CD146 was lower in the EDK cell line when compared with H9-MSCs. This elevated expression of CD10 and reduced expression of CD146 was also seen in HFFs, suggesting a fibroblast-like phenotype for EDK cells. These findings indicated that while both hES-derived cell populations exhibited characteristic mesenchymal surface markers, differences in their CD profile suggested differences in their biological potential.

### H9-MSCs undergo differentiation to osteogenic and adipogenic fates, while EDK cells do not show multilineage differentiation potential

**Figure 2:**
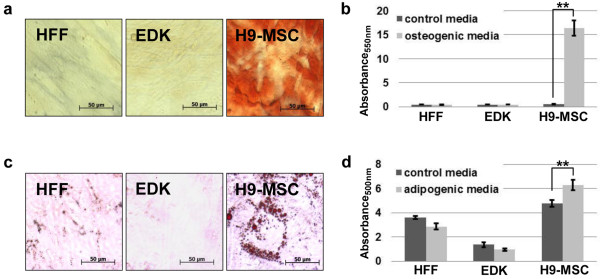
**Adipogenic and osteogenic differentiation of hES-derived mesenchymal cells**. **(a) **Analysis of osteogenic activity of EDK, H9-MSC, and human foreskin fibroblasts (HFF). Osteogenic differentiation was induced using the β-glycerophosphate method, after four weeks of differentiation, calcium deposition was stained with alzarin red. Bars, 50 μm. **(b) **Quantification of alizarin red staining (t-test: **P < 0.01). Results are expressed as the mean +/- standard deviation (SD) of three independent experiments and three technical replicates per experiment. **(c) **Analysis of adipogenic activity of EDK, H9-MSC, and HFF. Adipogenic differentiation was induced using the 3-isobutyl-1-methylxanthine (IBMX) method, and after four weeks of differentiation the lipid vesicles were stained with oil red O. Bars, 50 μm. **(d) **Quantification of oil red O staining (t-test: **P < 0.01). Results are expressed as the mean +/- SD of three experiments and three technical replicates per experiment.

To assess the biological potential of the EDK and H9-MSC populations, we performed osteogenic and adipogenic differentiation assays. After four weeks under differentiation conditions, H9-MSCs cultures contained calcium deposits as detected by alizarin red (Figures 2a and 2b), and oil red-O-positive lipid droplets (Figures 2c and 2d). In contrast, EDK did not show this differentiation potential as cells did not form bone or fat (Figure 2). These findings demonstrated that EDK and H9-MSC cell lines had divergent cell fates, as seen by their potential to form osteoblasts and adipocytes. Although H9-MSCs manifested properties of MSC-like progenitors, absence of multilineage differentiation potential in EDK cells suggested that these cells may have underwent developmental restriction to fibroblast lineage fate [10,24].

### EDK cells demonstrate fibroblast function characterized by their support of the normal development of 3D human skin equivalents

**Figure 3:**
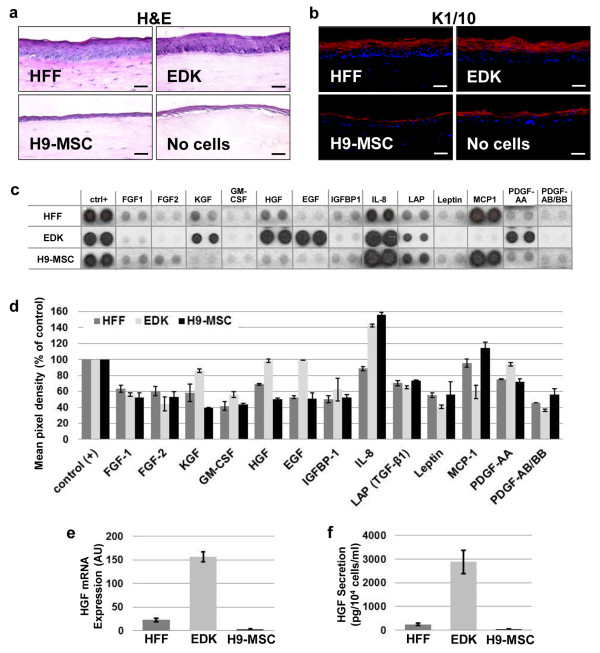
**EDK cells but not H9-MSC promote epidermal morphogenesis in HSE tissues**. **(a)**: Tissue morphology of human skin-equivalent (HSE) tissues constructed using human foreskin fibroblasts (HFF), EDK, H9-MSC, or without cells as analyzed by H&E staining. **(b) **The expression pattern of the epidermal differentiation marker keratin 1/10 was analyzed by immunofluorescence (red). Bars, 50 μm. **(c) **Comparison of the secretory profile of EDK, H9-MSC, and HFF cells by antibody-based cytokine array **(d) **Histogram profiles generated by quantifying the mean spot pixel densities by ImageJ from the array membrane shown above. The data are presented as percentages of the respective positive controls. **(e) **Hepatocyte growth factor (HGF) mRNA expression in HFF, EDK, and H9-MSC by real-time RT-PCR. Data are normalized to GAPDH, and all results are expressed as the mean +/- standard deviation (SD) of three experiments and three technical replicates per experiment. **(f) **Levels of HGF in supernatants from HFF, EDK, and H9-MSC monolayer cultures by ELISA. Data are normalized per 10^4 ^cells and all results are expressed as the mean +/- SD of three experiments and three technical replicates per experiment.

As the paracrine cross-talk between fibroblasts and epithelial cells is required for the development of stratified epithelial tissue, we next incorporated EDK or H9-MSC into HSEs as a functional assay for their capacity to support normal skin development. EDK or H9-MSC were embedded into collagen gels and allowed to contract the gel for seven days. Normal NHK were then seeded onto the surface of collagen gels, and tissues were grown at an air-liquid interface for an additional seven days to compare their capacity to form a multi-layered epithelium. HSEs constructed with HFFs or no cells embedded into collagen gels served as positive and negative controls, respectively. Morphological analysis revealed that EDK cells promoted epithelial tissue development in a manner similar to HFFs, while tissues grown in the presence of H9-MSC did not support normalization of the overlying epithelium (Figure 3a). HSEs constructed with EDK cells developed a fully-stratified, multi-layered epithelium that was well-differentiated and demonstrated tissue architecture similar to epithelial tissues generated with control HFF cells. In contrast, tissues populated with H9-MSCs generated a thin and poorly-developed epithelium that was similar to tissues generated in the absence of any fibroblast support. The characteristic supra-basal distribution of keratin 1/10, a marker of differentiated keratinocytes, in both EDK and HFF containing tissues confirmed the capacity of EDK cells to promote normal epithelial morphogenesis and differentiation (Figure 3b).

As efficient development of HSEs is dependent on paracrine support from connective tissue fibroblasts, as has been previously shown for mature fibroblasts [3-6], we used a panel of growth factors and cytokines known to function as mediators of epithelial cell growth to compare the secretory profile of EDK, H9-MSC, and HFF cells. Supernatants from EDK, H9-MSC, and control HFF cultures containing equal cell numbers were harvested and assayed using an antibody-based cytokine array (Figures 3c and 3d). This analysis revealed that EDK cells produced elevated levels of epidermal growth factor (EGF), granulocyte-macrophage colony-stimulating factor (GM-CSF), platelet-derived growth factor-AA (PDGF-AA), KGF, and HGF when compared with both H9-MSC and HFF cells. In contrast, H9-MSC produced higher levels of IL-8, monocyte chemotactic protein-1 (MCP-1), and PDGF-AB/BB than EDK or HFF cells. Notably, the level of HGF, known to be essential for skin development and repair [5,6] was elevated in both EDK and HFF cells but not in H9-MSCs. Using real-time PCR and ELISA, we detected a 10-fold elevation of HGF expression and secretion in EDK cells compared with HFF and a 100-fold elevation when compared with H9-MSC (Figures 3e and 3f). These findings indicated that the biological potential of hES-derived mesenchymal cells was not uniform. Although H9-MSCs could differentiate to osteogenic and adipogenic lineages, this cell line did not provide the paracrine support needed for epithelial development or maturation. In contrast, EDK cells demonstrated properties similar to dermal fibroblasts (HFF) based on their capacity to direct epithelial morphogenesis in an *in vivo*-like tissue environment.

### EDK cells accelerate the rate of wound healing in 3D human skin equivalents linked to their HGF production

**Figure 4:**
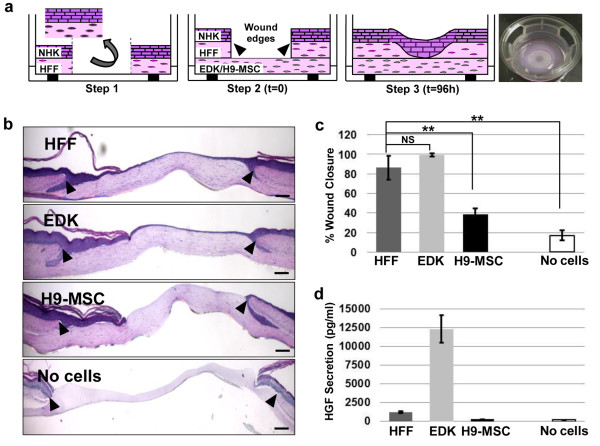
**EDK cells accelerate re-epithelialization of wounded HSEs**. **(a) **Schematic of 3D tissue model of wound re-epithelialization. In step 1, a full-thickness wound is generated by excising a human skin-equivalent (HSE). In step 2, the wounded HSE is placed on a second, contracted collagen gel populated with EDK, H9-MSC, human foreskin fibroblasts (HFF), or constructed without cells. In step 3, keratinocytes (NHK) undergo migration to close the wound gap and restored epithelial integrity. The far right panel is an image of six-well insert containing HSE 96 hours after wounding. **(b) **Representative morphology of wounded tissues constructed with HFF, EDK, H9-MSC, or without cells 96 hours after wounding (black arrows demarcate the initial wound edges). Bars, 200 μm. **(c) **EDK showed a rate of wound closure similar to tissues in which HFF were incorporated. The degree of re-epithelialization was significantly lower in H9-MSC and no cells-containing tissues as compared with HFF (t-test: **P < 0.01). **(d) **Levels of hepatocyte growth factor (HGF) in supernatants of wounded tissues 96 hours after wounding as measured by ELISA. HFF- and EDK-containing tissues produced higher levels of HGF as compared with H9-MSC-containing tissues or tissues constructed without cells. All results are presented as the mean +/- standard deviation of three independent experiments and three technical replicates.

We used our previously-developed 3D tissue model of cutaneous wound healing [22,23] to determine whether EDK and H9-MSCs could also modulate the re-epithelialization of wounded epithelia. To test this, HSEs constructed using HFF were wounded with a 4 mm punch and placed on the surface of a contracted collagen gel populated with either HFF, EDK, H9-MSCs, or without cells (see schematic in Figure 4a). HFFs have previously been shown to enable complete re-epithelialization [23,25] and serve as a positive control, while collagen gels generated without any cells were used as a negative control for re-epithelialization. Ninety-six hours after wounding, the wound bed was partially or completely covered by a migrating epithelial tongue towards the central area of the wound bed and by a more stratified epithelium toward the wound margins (Figure 4b). The degree of re-epithelialization following wounding was measured by calculating the distance separating the two epithelial tongues and was normalized to the distance between the initial wound margins and expressed graphically as the percentage of wound closure (Figure 4c). In general, EDK cells showed nearly complete or complete wound closure that was similar to tissues in which HFF were incorporated (HFF = 86 ± 12% and EDK = 99 ± 1.6%). In contrast, H9-MSCs-harboring tissues and the tissues constructed without any fibroblast support showed a significantly lower degree of re-epithelialization compared with HFF (H9-MSC = 39 ± 5.5% and no cells = 17 ± 5%, t-test: *P *= 0.003 and *P *= 0.0007, respectively; Figure 4c). To test whether degree of wound re-epithelialization was linked to HGF levels, HGF concentrations in supernatants from wounded HSEs were measured by ELISA. Quantification of HGF production revealed that both HFF- and EDK-containing tissues produced significantly higher levels of HGF (5-fold and 50-fold higher, respectively) than H9-MSC-containing tissues or in tissues without cells in their stroma (Figure 4d). These results imply that HGF-mediated paracrine signalling was responsible, at least in part, for the wound re-epithelialization mediated by EDK cells.

### Suppression of HGF production impairs the ability of EDK cells to promote wound healing in 3D human skin equivalents

**Figure 5:**
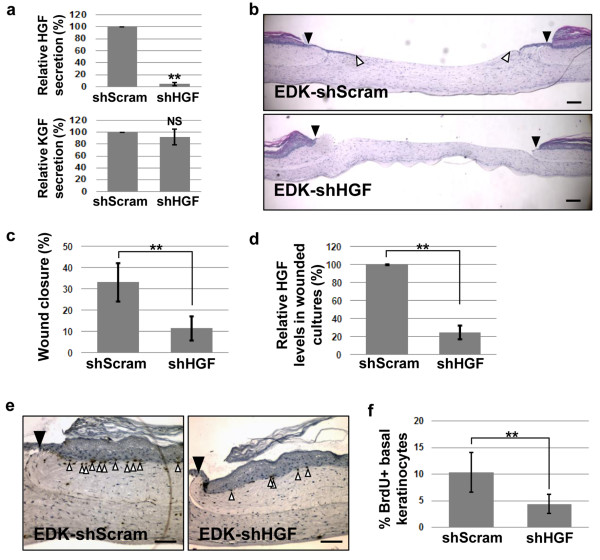
**Suppression of HGF production impairs EDK-mediated re-epithelialization of wounded HSEs**. **(a) **Efficiency of shRNA knockdown of hepatocyte growth factor (shHGF) in EDK cells relative to a scrambled, shRNA control (shScram) as measured by ELISA. Secretion of hepatocyte growth factor (HGF; top panel) was reduced 95% relative to shScram (t-test: **P < 0.01), secretion of KGF (lower panel) was not affected by shHGF. Data are normalized to shScram and all results are expressed as the mean +/- standard deviation (SD) of three experiments and three technical replicates per experiment. **(b) **Representative morphology of wounded tissues constructed with EDK-shHGF and EDK-shScram cells 72 hours after wounding (black arrows demarcate the initial wound edges; white arrows demarcate the tip of epithelial tongues). Bars, 200 μm. **(c) **The degree of re-epithelialization was significantly lower in tissues containing EDK-shHGF cells as compared with EDK-shScram (t-test: **P < 0.01). **(d) **Relative levels of HGF in supernatants of wounded tissues 72 hours after wounding as measured by ELISA. Tissues containing EDK-shHGF cells produced significantly lower levels of HGF as compared with EDK-shScram (t-test: **P < 0.01). **(e) **Proliferation of basal keratinocytes in tissues containing EDK-shHGF and EDK-shScram cells was analyzed using immunoperoxidase staining with anti-BrdU antibody 72 hours after wounding (black arrows demarcate the initial wound edges; white arrows demarcate the BrdU-positive cells). **(f) **Quantification of BrdU-positive basal keratinocytes. Tissues containing EDK-shHGF demonstrated significantly lower percentage of proliferating basal keratinocytes compared with EDK-shScram (t-test: **P < 0.01). Bars, 100 μm. All results are presented as the mean +/- SD of three independent experiments and three technical replicates.

To confirm that HGF secretion is necessary to optimally direct the re-epithelialization of cutaneous wound mediated by EDK cells, levels of HGF secreted from EDK cells were significantly reduced by introducing a lentiviral shRNA construct that targeted HGF mRNA (shHGF). Efficiency of shHGF in EDK cells was measured relative to a scrambled, shRNA control (shScram) by ELISA and showed a 95% reduction in secretion of HGF from EDK cells (Figure 5a, top panel). This shHGF lentiviral construct did not alter secretion of other growth factors, such as KGF (Figure 5a, lower panel). The effect of decreased HGF secretion on the capacity of EDK cells to promote re-epithelialization of wounded HSEs was examined by incorporating EDK-shHGF and EDK-shScram into 3D tissue model of cutaneous wound repair. Tissue morphology and the degree of re-epithelialization following wounding were analysed 72 hours after wounding. Tissues constructed using EDK-shHGF cells showed a significantly lower degree of re-epithelialization compared with EDK-shScram (EDK-shScram = 33 ± 9% vs. EDK-shHGF = 11.6 ± 5.8%, t-test: *P *= 0.017; Figures 5b and 5c). HGF levels in supernatants from wounded HSEs were measured by ELISA and showed roughly 85% reduction of HGF levels in cultures containing EDK-shHGF (Figure 5d). To further characterize the phenotype of wounded epithelial tissues in the presence of EDK-shHGF and control EDK-shScram, we assayed proliferation of keratinocytes in the basal layer of wound margins using a BrdU-incorporation assay (Figure 5e). Tissues harboring EDK-shHGF demonstrated a significantly lower percentage of proliferating basal keratinocytes compared with EDK-shScram (EDK-shScram = 10 ± 3.5% and EDK-shHGF = 4.4 ± 1.8%, t-test: *P *= 0.004; Figure 4f). These results support the known function of HGF as a mediator of epithelial cell proliferation in restoring epithelial integrity following wounding [6]. This suggested that hES-derived EDK cells stimulated wound re-epithelialization by providing paracrine signals, such as HGF, that is needed to direct the repair of a cutaneous wound.

## Discussion

Our comparative characterization of two hES-derived cell lines (EDK and H9-MSC) that were differentiated under different conditions has revealed that both cell lines exhibited similar morphology and cell surface marker expression indicative of a mesenchymal phenotype [19]. However, although EDK cells showed some phenotypic overlap with H9-MSC and adult MSCs [19] in two dimensional (2D) monolayer culture, their functional properties in engineered 3D tissues and the absence of the multilineage differentiation potential seen in H9-MSC provided evidence of their fibroblast lineage commitment. Thus, we have demonstrated that engineered, 3D tissues can be used as a sensitive assay to characterize the biological potency of hES-derived fibroblasts. Incorporation of hES-derived cells into tissues that mimic their *in vivo *counterparts enables a more complete determination of their lineage commitment and phenotype than conventional, monolayer cultures. We have previously generated a fibroblast-like cell line that partially supported a 3D tissue whose surface cells were hES-derived cells with ectodermal features [18]. We now extend those studies using an hES-derived cell line that produced elevated levels of HGF to provide paracrine support for tissue development and repair that mimics the functional features of dermal fibroblasts. We have shown the suppression of HGF production in EDK cells is linked to a significant decrease in epithelial proliferation and repair, suggesting that these cells mediate epithelial tissue phenotype through a paracrine support. In contrast, a hES-derived cell line that manifests multilineage differentiation, but very low HGF levels (H9-MSC) was unable to support epithelial tissue development and repair.

Despite the critical impact of fibroblasts on tissue morphogenesis, homeostasis, and repair, gaps in our understanding of fibroblast lineage development has made the identification and reproducible isolation of specific fibroblast populations particularly challenging [8,9]. In this light, we expect that our findings will help address a central challenge facing clinical application of cells with properties of dermal fibroblasts for regenerative medicine by developing an efficient approach to reproducibly procure these cells from pluripotent stem cells, in a way that offers predictable and effective tissue outcomes upon their therapeutic use [1,2].

In previous studies, MSC-like cells expressing α-smooth muscle actin (α-SMA) have been derived from hES cells [26,27] and fibroblast-like cells isolated from hES cells have been used as autogenic feeders to support the culture of undifferentiated hES cells [14-17]. In addition, we have found that surface marker profile of EDK and H9-MSC cell lines is similar to those expressed on MSCs previously derived from hES cells using alternative differentiation conditions to those described in our study [10-13]. However, MSC-like cells derived in these previous studies were not grown in 3D tissue microenvironments that could provide a more complete functional characterization of their identity as fibroblasts. As the use of cell surface markers to distinguish MSCs from cells restricted to fibroblast lineage fate is limited, tissue-based, functional readouts are essential to predict the potency of hES-derived cells differentiated into fibroblast lineages [24,28]. As it is possible that EDK cells represent a heterogeneous mixture of fibroblasts and a smaller number of cells that retain MSC-like features, their future therapeutic use may require further characterization of fibroblast subpopulations. This approach has recently been demonstrated to improve the specificity of MSCs differentiated from hES cells in ways that can select for reproducible and clinically-applicable cells [11]. In addition, it remains unclear what factors may direct the differentiation of EDK cells to a fibroblast fate.

Transplantation of adult-derived MSCs has previously been shown to promote tissue regeneration and to improve wound repair *in vivo *[29-32]. Increasing evidence suggests that this repair potential is linked to the ability of MSCs to secrete paracrine-acting factors that can induce changes in their tissue microenvironment to guide healing [33,34]. We found that EDK cells secreted a broad repertoire of growth factors and cytokines linked to a variety of paracrine-mediated tissue responses, including stimulatory, paracrine factors known to accelerate the healing of epithelial wounds. Specifically, we found that this augmented re-epithelialization of wounded keratinocytes was linked to the production of HGF by EDK cells, as the diminished production of this growth factor upon shRNA infection significantly decreased tissue re-epithelialization. As HGF is known to be induced in stromal cells in response to injury [5,6], directed differentiation of hES-derived fibroblasts may allow these cells to be adapted for cell therapy and cutaneous regeneration. Beyond this, a decline in fibroblast numbers or function has been implicated in both skin aging and cancer development, because the age-related accumulation of senescent fibroblasts is thought to create a microenvironment that promotes squamous cell carcinoma progression [35-37]. As restoration of growth factor production in fibroblasts may decrease cancer risk and sustain tissue homeostasis, the development of future cell-based treatments using hES-derived fibroblasts can potentially augment repair and improve overall tissue health. We expect that our findings will enable future studies aimed at clarifying factors regulating fibroblast development and will help identify progenitor cells that give rise to these cells in human skin and other tissues.

## Conclusions

A central challenge that must be overcome before therapeutic application of hES-derived cells is the development of reliable and sensitive methods to evaluate their safety and efficacy [1,2]. Although hES cells can undergo directed differentiation to numerous cell types, progress towards their clinical application has been limited by the lack of tools and platforms to evaluate their stability in an *in vivo*-like tissue context. It is therefore critical to fully-characterize the properties of differentiated cells derived from hES cells by developing engineered, pre-clinical tissue models that will better predict their behavior following future therapeutic transplantation to humans. As we have shown that fibroblasts derived from hES cells support the normal development of HSEs, our study is an important step towards determining the biological potency of clinically-relevant, functional cell types derived from hES cells.

## Abbreviations

2D: two-dimensional; 3D: three dimensional; DMEM: Dulbecco's modified eagle medium; ELISA: enzyme-linked immunosorbent assay; FACS: fluorescence-activated cell sorting; FBS: fetal bovine serum; hES cells: human embryonic stem cells; hiPS cells: human induced pluripotent stem cells; HFF: human foreskin fibroblasts; HGF: hepatocyte growth factor; HSE: human skin equivalent; IL: interleukin; MSC: mesenchymal stem cells; MEF: mouse embryonic fibroblasts; NHK: normal human keratinocytes; PBS: phosphate buffered saline; RT-PCR: reverse-transcription polymerase chain reaction; SD: standard deviation.

## Competing interests

The authors declare that they have no competing interests.

## Authors' contributions

YS participated in conception and design, collection and assembly of data, data analysis and interpretation, and drafted the manuscript. KJH participated in provision of study material, data analysis and interpretation, and final approval of manuscript. MWC participated in data analysis and interpretation, and final approval of manuscript. MM participated in collection and assembly of data and final approval of manuscript. SD participated in provision of study material and final approval of manuscript. CKK participated in collection and assembly of data and final approval of manuscript. LD participated in provision of study material and final approval of manuscript. CE participated in collection and assembly of data and final approval of manuscript. JAG participated in conception and design, data analysis and interpretation, and helped to draft the manuscript.
